# *QuickStats:* Percentage* of Adults Aged ≥18 Years Who Walked for Transportation and Walked for Leisure in the Past 7 Days,^†^ by Urban-Rural Status^§^ — United States, 2022

**DOI:** 10.15585/mmwr.mm7328a4

**Published:** 2024-07-18

**Figure:**
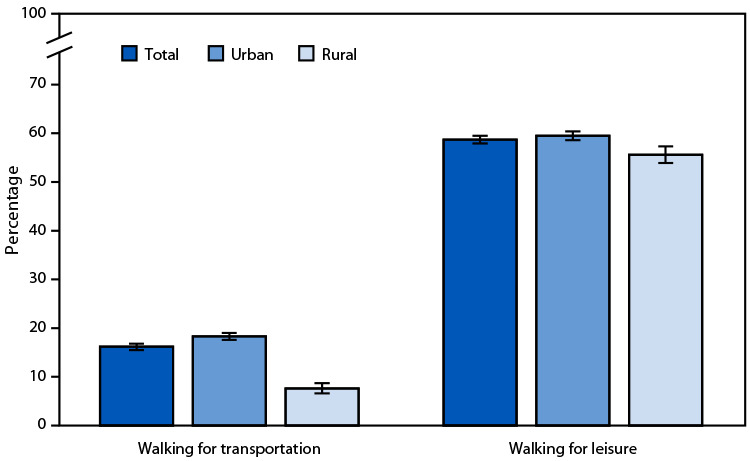
In 2022, among adults aged ≥18 years, 16.2% walked for transportation in the past 7 days, and 58.7% walked for leisure in the past 7 days. Urban residents were more likely than rural residents to walk for transportation and leisure.

**Supplementary Table:** https://stacks.cdc.gov/view/cdc/157542

**Source:** National Center for Health Statistics, National Health Interview Survey, 2022. https://www.cdc.gov/nchs/nhis/index.htm

For more information on this topic, CDC recommends the following link: https://www.cdc.gov/nccdphp/dnpao/features/getting-more-active-minutes/index.html

